# Histone Deacetylases Inhibitors in Neurodegenerative Diseases, Neuroprotection and Neuronal Differentiation

**DOI:** 10.3389/fphar.2020.00537

**Published:** 2020-04-24

**Authors:** Surabhi Shukla, Babu L. Tekwani

**Affiliations:** ^1^ Department of Pharmaceutical Sciences, College of Pharmacy, Larkin University, Miami, FL, United States; ^2^ Division of Drug Discovery, Department of Infectious Diseases, Southern Research, Birmingham, AL, United States

**Keywords:** histone deacetylase, neurodegenerative disease, histone deacetylases inhibitors, neuritogenesis, neuronal differentiation, neuroprotection

## Abstract

Histone deacetylases (HADC) are the enzymes that remove acetyl group from lysine residue of histones and non-histone proteins and regulate the process of transcription by binding to transcription factors and regulating fundamental cellular process such as cellular proliferation, differentiation and development. In neurodegenerative diseases, the histone acetylation homeostasis is greatly impaired, shifting towards a state of hypoacetylation. The histone hyperacetylation produced by direct inhibition of HDACs leads to neuroprotective actions. This review attempts to elaborate on role of small molecule inhibitors of HDACs on neuronal differentiation and throws light on the potential of HDAC inhibitors as therapeutic agents for treatment of neurodegenerative diseases. The role of HDACs in neuronal cellular and disease models and their modulation with HDAC inhibitors are also discussed. Significance of these HDAC inhibitors has been reviewed on the process of neuronal differentiation, neurite outgrowth and neuroprotection regarding their potential therapeutic application for treatment of neurodegenerative diseases.

## Introduction

Reversible histone acetylation and deacetylation functions play a key role in gene regulation. The process of acetylation and deacetylation are accelerated by enzymes Histone acetyl transferases (HATs) and Histone deacetylases (HDACs), respectively (Icardi et al., 2012; Rousseaux and Khochbin, 2015). Small molecule inhibitors of HDACs play important role in transcription regulation by changing the steady state of cells towards hyperacetylation. In neurodegenerative disease, the histone acetylation homeostasis is significantly compromised, shifting towards hypoacetylation (Saha and Pahan, 2005). The histone hyperacetylation produced by direct inhibition of HDACs leads to neuroprotective actions. This review presents a background on protein lysine acetylation and deacetylation functions, histone acetylation and deacetylation, HATs and HDACs and HDAC inhibitors (HDACi). The review particularly emphasizes details on HDAC inhibitors and their potential therapeutic application in various neurodegenerative diseases. The importance of HDAC inhibitors in neuronal differentiation and neuroprotection is also discussed.

## Acetylome: Protein Lysine Acetylation and Deacetylation Functions

Post-translational modifications are prominent, important and necessary processes for transformation of raw protein molecules into functional entities. The acetylation of proteins at specific lysine residues by acetyltransferases enzymes has emerged as a biologically related regulatory modification like phosphorylation (Kouzarides, 2000). The acetyl transferases catalyze the transfer of an acetyl group from acetyl Co-A to α-amino group of the amino terminal residues or to the ϵ-group of specific lysine residue (Allis et al., 2007). Deacetylation, the reverse reaction of removal of acetyl group from acetyl lysine residues, is accomplished by another group of enzymes named deacetylases. Lysine protein acetylation was first identified in histones many years ago. Lysine acetyl transferases (KATs) and lysine deacetylases (KDACs) are often referred to as histone acetyl transferases (HATs) and histone deacetylases (HDACs) (Yang and Seto, 2008). A variety of proteins undergo acetylation process including histones and non-histone proteins. More than 80 transcription factors are known for Lysine acetylation, including a large number of nuclear regulators and several cytoplasmic proteins (Glozak et al., 2005 *)*. Posttranslational modifications play a very significant role in a variety of cellular and biological process. Some of the prominent cellular process controlled by the lysine protein acetylation/deacetylation include chromatin modification and transcription, gene silencing, cellular differentiation, cell cycle progression, DNA replication, DNA repair (Choudhary et al., 2009; Aksnes et al., 2015) and apoptosis (Lebel et al., 2010). Most of the intermediate enzymes in the metabolic pathways are acetylated. Acetylation of these enzymes regulates cellular metabolism by directly affecting their stability and functions (Zhao et al., 2010). Lysine protein acetylation also plays a key role in p53 functions and interaction and stabilization of microtubules.

## Histone Acetylation and Deacetylation

**Figure 1:**
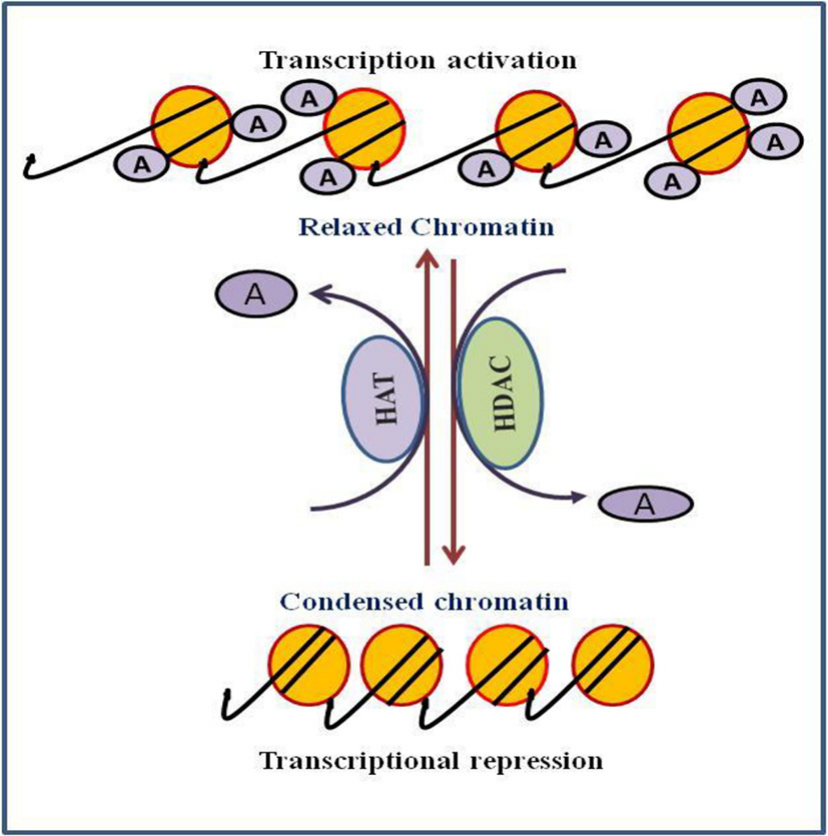
Role of histone acetylation and deacetylation in process of transcription regulation.

Chromatin is a complex structure in which DNA and histones are bundled within the nucleus of the cell. The vital component of the chromatin is the nucleosome, which is composed of octamers of the four essential histones (H3, H4, H2A, and H2B). Each histone octamer is wrapped around with 147 base pairs of DNA (Kouzarides, 2007). Chromatin is a very active structure as it responds to various signals and control functions of the DNA. Histone modifications are the main components that regulate DNA functions. Histones are well known to undergo a variety of post-translational modifications, namely phosphorylation, acetylation, methylation, and ubiquitination (Bannister and Kouzarides, 2011). Histone acetylation is a prominent and most important post-translational modifications that regulates gene expression. Acetylation and deacetylation are highly dynamic process and catalyzed by the interplay of two opposing enzymes histone acetyl transferases (HATs) and histone deacetylases (HDACs) (**Figure 1**). HATs catalyze the addition of an acetyl group at the lysine residue while HDACs catalyze removal of acetyl group from epsilon amino group of the lysine side chain of histone. Acetylation of histone tails neutralizes their positive charge resulting into the loosening of histones and the associated DNA, thereby providing the loose chromatin structure that is accessible to the transcription factors and thus promoting transcriptional activation (Parthun, 2007). Conversely, deacetylation of histone favors chromatin compaction. Generally, hyperacetylation of histone is associated with transcriptional activation, whereas hypoacetylation is associated with transcriptional repression (Hsieh et al., 2004).

## Histone/Lysine Acetyl Transferases (HATs or KATs)

**Figure 2:**
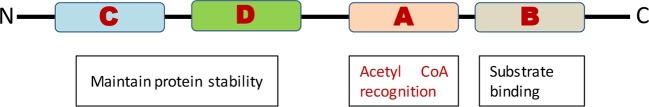
Conserved motifs of GNATs and their functions.

In humans, there are about 30 known HAT/KATs, which have been grouped into five distinct families based on the functional and structural similarity of their catalytic domains (Dekker and Haisma, 2009). The HATs arrange in distinct multi-subunit complexes, which provides them the ability to regulate their properties like substrate specificity and selectivity like targeting to specific loci and selectivity to access non-target proteins (Berndsen and Denu, 2008; Wapenaar and Dekker, 2016). GCN5 related N-Acetyl transferases (GNATs) have derived their name from bearing similarity to yeast GCN5 (general control nonderepressible-5) enzymes, having GCN5, and its relative and some distantly related HATs like Hat1, Elp3, and Hpa2. GNATs are cluster of four conserved motifs (A–D) within the catalytic domain HAT domain (**Figure 2**). Motif A is the most highly conserved among all four motifs, and contains an Arg/Gln-X-X-Gly-X-Gly/Ala sequence that is important for acetyl-CoA recognition (Roth et al., 2001). The chromodomain or bromodomain in GNATs are involved in their binding with methylated and acetylated lysine respectively (Neuwald, 1997). The MYST families of HATs are initially called after their pioneer family members MOZ, Ybf2, Sas2, and Tip60. The MYST HATs plays a very important role in posttranslational modification of histones and have a characteristic domain that contains acetyl CoA binding motif and a zinc finger motif (Avvakumov and Côté, 2007). The other families are global co-activators nuclear receptor co-activators and divers. The global co-activator includes p300 and CREB binding Protein. Nuclear receptor co-activators include steroid receptor co-activator (SRC-1), ACTR, and (Transcriptional intermediary factors) TIF2. The divers include TAFII 250 [TATA binding protein (TBP)—associated factor] and P160 (Sadoul et al., 2011).

## Histone Deacetylases (HDACs)

Histone deacetylases are enzymes that remove an acetyl group from histones leading to compact chromatin structures. HDACs are part of co-repressor complexes. The HDACs are well known to control multiple vital biological processes such as cell proliferation, differentiation and development by binding to many transcription factors and transcription co-regulators (Haberland et al., 2009).

**Figure 3:**
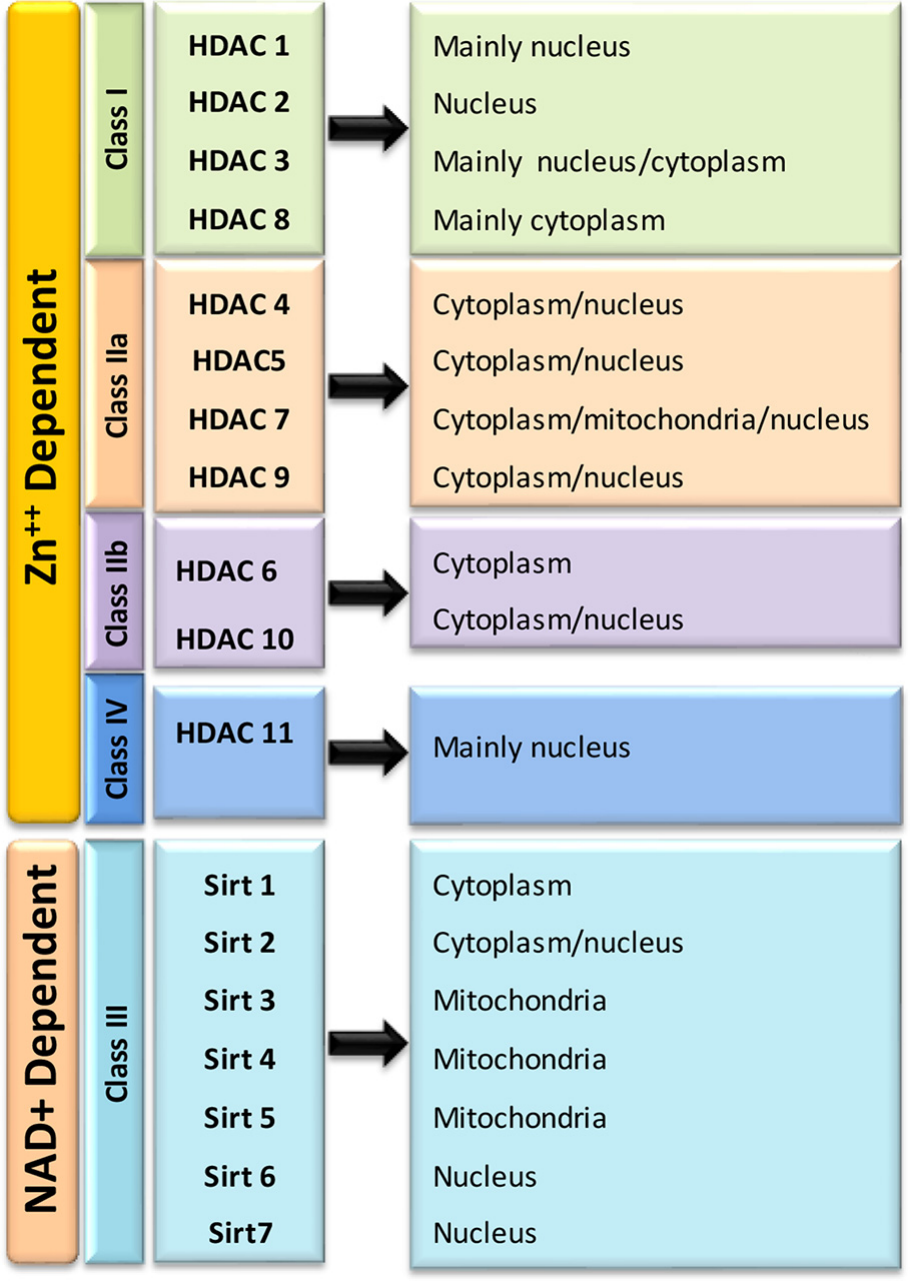
Classification of Histone Deacetylases (HDACs) and their cellular localization.

Till date, total eighteen mammalian HDACs are well characterized and grouped into four major classes based upon their homology to yeast histone deacetylases (**Figure 3**). In Class 1, HDACs 1, 2, 3, and 8 are clustered based on their similarity to yeast transcriptional regulator, reduced potassium deficiency 3 (Rpd3) (De Ruijter et al., 2003). The Class I HDACs are predominantly localized in the nucleus. HDACs 1, 2, and 3 are shown to be involved in cell proliferation and death (Wiech et al., 2009) while HDAC 8 is found to be expressed in smooth muscle and plays a key role in muscle contractility. The Class II deacetylases share similarity with yeast Histone Deacetylase 1 (HD1) proteins and are further grouped into class IIa and Class IIb based on their structural similarity. The Class IIa includes HDACs 4,5, 7, and 9, while Class IIb includes HDAC 6 and 10. The classes II HDACs have been found to travel back and forth between the nucleus and cytoplasm and seem to have a role in tissue specific developmental activities (Bjerling et al., 2002). Class IV includes only one atypical HDAC isoform 11. It is well expressed in the kidneys, testis and brain. HDAC11 has been identified to deacetylate histones along with some non-histone proteins, namely a nuclear hormone receptor, transcription factors and cytoskeletal elements (Yang and Seto, 2008; Haberland et al., 2009). The Class I, II, and, IV HDACs are zinc-dependent as a cofactor and known as the classical HDACs. Class III HDACs, commonly called sirtuins (SIRT 1-7), are typically different from the classical HDACs. These NAD-dependent HDACs are similar to yeast SIR 2 (Silent Information Regulators 2) (Wiech et al., 2009). Sirtuins are involved in regulation of metabolism, stress response and aging (Michan and Sinclair, 2007).

## HDAC Inhibitors (HDACi)

**Figure 4:**
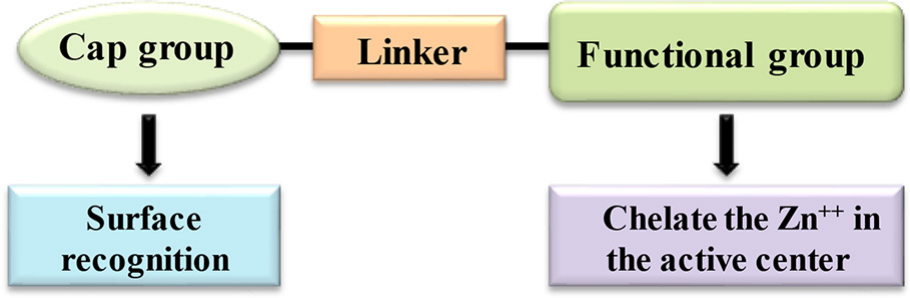
Basic pharmacophore structure of HDAC inhibitors.

HDACi are small molecule natural or synthetic inhibitors of HDAC enzymes. These compounds vary in structures, selectivity and biological activities. HDACi pharmacophore is typically consisting of a metal-binding moiety or functional group, a capping group and a linker. The metal binding group, facilitates the catalytic metal binding to the active site of HDAC. The capping group interacts with the amino acids at lysine binding site while the linker having the structural similarity to the carbon chain present in the acetyl-lysine substrate, connects the metal binding and capping group for interaction with the HDAC active site (Finnin et al., 1999) (**Figure 4**). Recently, alternative strategies have been also suggested for achieving selective inhibition of HDAC that relies on perturbation of protein-protein interactions vital for the HDAC activity, rather than a traditional approach of the zinc ion chelation at the active site (Maolanon et al., 2016).

**Figure 5:**
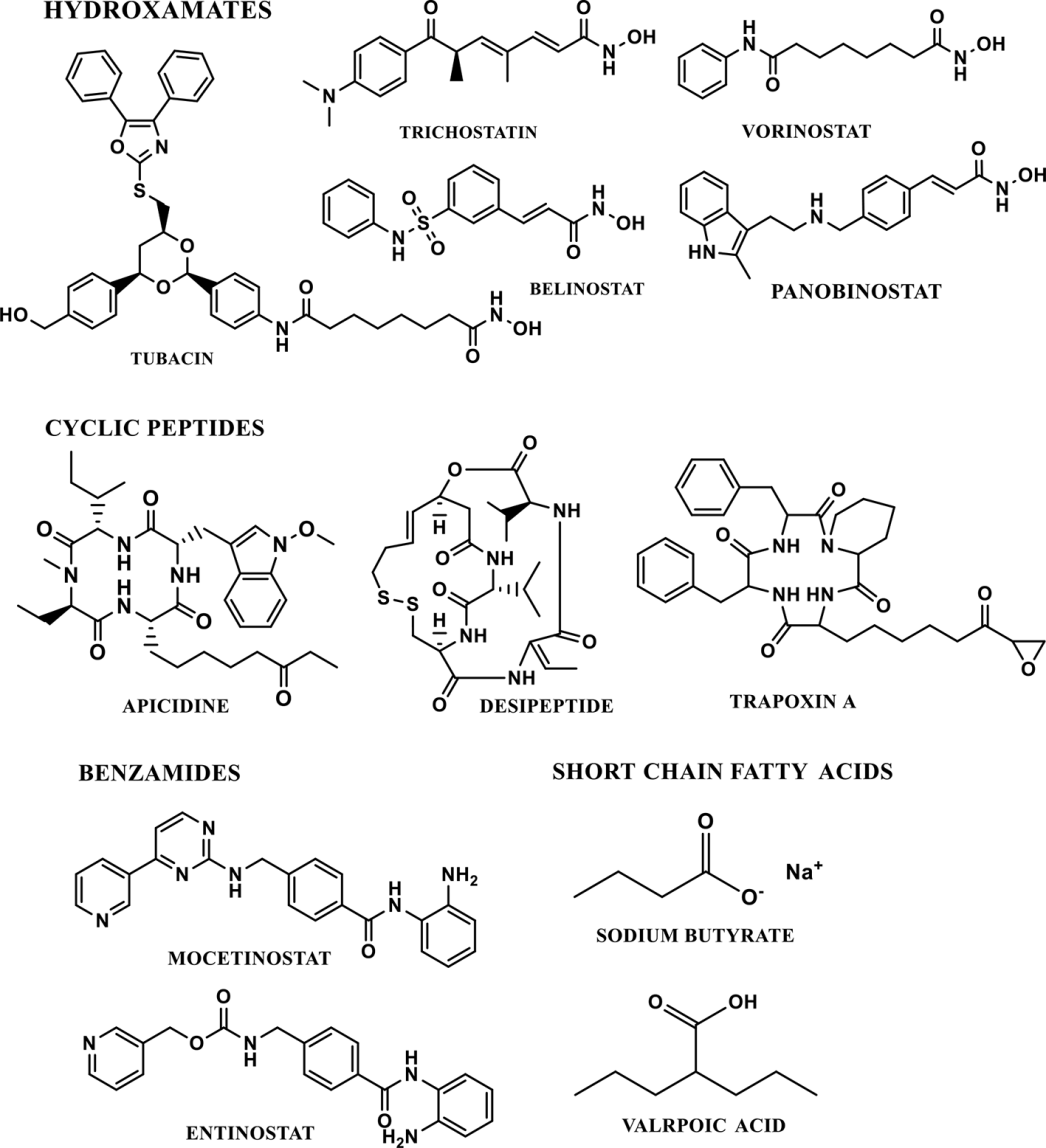
Chemical classes and structures of selected HDAC inhibitors.

The majority of HDAC inhibitors have been reported and their potential therapeutic applications have been discussed (Butler and Bates, 2006; Marks, 2010). HDACi have been classified into four major classes, based on the chemical nature of the inhibitor (**Figure 5**). A) Hydroxamates (e.g. Vorinostat (SAHA), Belinostat (PXD101), Panobinostat (LBH589) and Trichostatin A. The hydroxamate HDACi are non-specific and show activity against class I and class II HDACs. Trichostatin A and vorinostat are pan-HDAC inhibitors of all zinc-dependent HDACs and are reported to cross the blood brain barrier. B) The cyclic peptide HDACi includes depsipeptides and trapoxin. In *in-vitro* assays, cyclic peptides are active at nanomolar concentrations (Marks, 2010). C) The benzamide HDACi include entinostat (MS-275) mocetinostat, and D) Short chain fatty acids HDACi include sodium butyrate and valproic acid (Marks, 2010). The short chain fatty acids HDACi could also cross the blood brain barrier, however they are relatively moderate HDACi (Butler and Bates, 2006). The majority of HDACi available are non-specific inhibitors of all the HDAC isoforms. These are generally referred as pan-HDAC inhibitors. TSA and vorinostat are an example of canonical pan-HDAC inhibitors that inhibit HDACs 1–9 with equivalent potency. Selective HDACi can be classified into either class-specific (inhibiting several isoforms within a single class) or isoform-specific HDAC inhibitor (selectively inhibiting a specific HDAC isoform).

Selective HDAC inhibitors are important for determining the molecular mechanism of functions of a specific HDAC isoform. Recently, significant attempts have been made towards the development of selective HDAC inhibitors. Tubacin, an HDAC inhibitor, selectively targets the HDAC6 and deacetylation of α-tubulin (Haggarty et al., 2003). Romidepsin (FK-228) is a cyclic tetrapeptide, which selectively inhibits HDAC 1 and 2 isoforms (Itoh et al., 2008). Apicidin, another cyclic tetrapeptide is a highly potent inhibitor of HDAC2 & 3(Khan et al., 2008). MS-275, a synthetic benzamide derivative, preferentially inhibits HDAC1 as compared to HDAC 2, 3 and 9. MS-275 exhibits little to no activity against HDAC 4, 6, 7, and 9 (Khan et al., 2008). Suramin inhibits human NAD+ dependent class III SIRT 1 and SIRT 2 activity (Trapp et al., 2007).

## HDACi as Potential Therapeutic Agents for Treatment of Neurological Disorders

In recent years, inhibition of HDAC activity with HDACi has attracted significant therapeutic attention. Earlier interests were mainly based on the development of HDACi as anticancer agents (Kazantsev and Thompson, 2008; Tsilimigras et al., 2018). Some HDAC inhibitors, like butyrate, trichostatin A (TSA), suberoylanilide hydroxamic acid (SAHA), MS-275, inhibit cell growth and stimulate cell differentiation in various *in-vitro* cancer models, including leukemia. SAHA (vorinostat, brand name-Zolinza) become the first HDACi approved for cutaneous T-cell leukemia by the FDA. Several HDACi are now in Phase I and Phase II clinical trials for cancer therapeutics (Marks and Xu, 2009). Some HDACi used in clinical trial for neurological/cancer and other conditions are summarized in **Table 1**. In recent years, the therapeutic interests in HDACi have extended to non-malignant conditions impacting the nervous system (Kazantsev and Thompson, 2008; Konsoula and Barile, 2012; Ziemka-Nalecz et al., 2018). Targeting HDACs with HDACi might have potential for treatment of neurological disorders such as Huntington's disease, Alzheimer's disease, amyotrophic lateral sclerosis, seizure disorders, spinal muscular atrophy, Rett syndrome, stroke, Fragile X syndrome, and Rubinstein-Taybi syndrome (Chuang et al., 2009). The HDACi also seem promising for several psychiatric disorders therapeutics like depression, drug addiction, schizophrenia, and anxiety disorders (Butler and Bates, 2006; Guidotti et al., 2009). The levels and activities of HATs and HDACs are finely balanced in neuronal cells under normal conditions (Saha and Pahan, 2005). In neurodegenerative disease, the histone acetylation homeostasis is greatly impaired, shifting towards hypoacetylation (Saha and Pahan, 2005). The histone hyperacetylation produced by direct inhibition of HDACs leads to neuroprotective actions. Besides, inhibition of HDACs and histone hyperacetylation, the neuroprotective effect of HDACi may also involve multiple mechanisms of action, involving activation of the kinase pathway by extrinsic signals (Hao, 2004) the suppression of pro-apoptotic factors (Kim et al., 2007) or microglial–mediated inflammation (Peng et al., 2005), as reported earlier for valproic acid. Therefore, HDACs show great potential as a cellular target for the treatment of neurological and psychiatric disorders. In preclinical treatment models, the HDACi have exhibited neuroprotective effects and stimulation of neurogenesis in traumatic brain injury and ischemia (Kim et al., 2009), restoration of memory and learning in traumatic brain injury in neurodegenerative mice (Dash et al., 2009), enhanced neuronal differentiation and synaptic plasticity (Vecsey et al., 2007) and exerted antidepressant-like effects (Schroeder et al., 2007). Vorinostat significantly reduced IFN- λ induced neurotoxicity of human astrocytes (Hashioka et al., 2012).

**Table 1:** Examples of HDAC Inhibitors in Clinical trials.

HDAC Inhibitors in Clinical Trials		
HDAC inhibitors	Disease/Cancer	Reference
Valproic Acid + Temolozide + Radiation therapy	High grade glioma/Brain tumor	https://ClinicalTrials.gov/show/NCT00302159
Vorinostat	Progressive recurrent Glioblastoma multiforme	https://ClinicalTrials.gov/show/NCT00238303
Vorinostat + Bortezomib	Progressive recurrent Glioblastoma multiforme	https://ClinicalTrials.gov/show/NCT00641706
Vorinostat	Acute Myeloid Leukemia	https://ClinicalTrials.gov/show/NCT00305773
Vorinostat	Low grade Non Hodgkins Lymphoma	https://ClinicalTrials.gov/show/NCT00253630
Vorinostat	Progressive prostate cancer	https://ClinicalTrials.gov/show/NCT00330161
Vorinostat+ Rituximab	Inoldent Non-Hodgkins Lymphoma	https://ClinicalTrials.gov/show/NCT00720876
Vorinostat (SAHA)	Primary Cutaneous T-Cell lymphoma	https://clinicaltrials.gov/ct2/show/NCT00958074
Belinostat (PXD101)	Advanced stage tumor of Thymus	https://ClinicalTrials.gov/show/NCT00589290
Belinostat (PXD101)	Myelodysplastic syndrome	https://ClinicalTrials.gov/show/NCT00357162
MS-275 + GMSF	Refractory/relapsed Acute Myeloid Leukemia or Acute Lymphocytic Leukemia	https://ClinicalTrials.gov/show/NCT00462605
Paobinostat (LBH859)	Refractory clear cell renal carcinoma	https://ClinicalTrials.gov/show/NCT00550277
Panobinostat + Lenalidomide and Dexamethasone	Multiple Myeloma	https://ClinicalTrials.gov/show/NCT01651039
Depsipeptide (Romidepsin) FK228	Cutaneous T-cell lymphoma	https://ClinicalTrials.gov/show/NCT00007345
Givinostat (ITF237) + hydroxyurea	Polycythemia vera	https://ClinicalTrials.gov/show/NCT00928707

Table provides a partial list of HDAC inhibitors in clinical trials as monotherapy and in combinations.

Treatment with HDAC inhibitors results in the hyperacetylation of chaperones including heat shock protein (HSP90, HSP70, HSP40, glucose-regulated protein 78 (GRP78) in turn affecting their function. Chaperones are proteins molecules that facilitate the folding and maturation of newly synthesized proteins and partially folded proteins in the cytosol and endoplasmic reticulum. Chaperons also play important role in preventing the aggregation of misfolded proteins (Rao et al., 2012). The mechanism of protection after cerebral ischemic events is not very clear; however, it has largely been attributed to HSP 70 chaperone functions that enhance cell survival by blocking protein aggregation (Giffard et al., 2004). There are other studies that show HSP 70 can also modulate inflammatory response. In the ischemic brain, treatment with HDAC inhibitors, markedly inhibited ischemia induced p53 overexpression and superinduced heat shock protein 70 (HSP70) (Ren et al., 2004; Faraco et al., 2006; Kim et al., 2007). Super induction of HSP 70 protein by HDAC inhibition may cause anti-inflammatory effects. A recent study, in a mouse transient middle artery cerebral occlusion model (tMCAO) model shows that HSP70 overexpression inactivates NF-κB by stabilizing a complex of HSP70-IκBα-NF-κB (Zheng et al., 2008).

The pathophysiology of Huntington's disease is closely linked to BDNF and HSP70 deficiency in affected brain regions. Class I and class II HDAC inhibitors regulate expression of BDNF and HSP70. Therefore, it is plausible that restoring BDNF and HSP70 to their normal levels is the molecular mechanism underlying the beneficial effects produced by HDAC inhibition in various HD models. (Zuccato et al., 2001; Hay et al., 2004; Tagawa et al., 2007).

The pathophysiology of Parkinson's disease is associated with aggregation of α-synuclein and progressive degeneration of dopaminergic neuron. In context to that, HDAC6 is reported to rescue polyglutamine-mediated neurodegeneration in an autophagy-dependent manner (Pandey et al., 2007). HDAC 6 senses ubiquitinated aggregates and subsequently activates the expression of chaperones, such as heat-shock protein Hsp70 and Hsp25 (Boyault et al., 2007). HDAC 6 plays important role in cellular function through its enzymatic and nonenzymatic actions (Seigneurin-Berny et al., 2001). The catalytic domains of HDAC6 promotes deacetylation of many non-histone proteins, such as HSP-90, a-tubulin, and cortactin, (Luxton and Gundersen, 2007; Valenzuela-Fernandez et al., 2008). The nonenzymatic action of HDAC6 is associated with ubiquitin binding domain that regulates the buildup of ubiquitinated misfolded proteins (Du et al., 2010; Du and Jiao, 2011). The results indicate that HDAC6 promotes cytoplasmic inclusion formation and facilitates autophagic degradation of these aggregates in the aggresome. Therefore, this action protects dopaminergic cells against a-synuclein toxicity and might have an important role in the suppression of Parkinson's disorder (Li et al., 2011). These findings were further confirmed by treatment of HDAC6 inhibitor tubacin to PC-12 cells over-expressing human mutant (A53T) α-synuclein (α-syn) and SH-SY5Y cells with MPP (+) that led to defects in aggresome formation accompanied by massive cell death in response to misfolded protein-induced stress (Su et al., 2011). A study in Alzheimer's disease models shows importance of HSP-70 and Glucose regulated protein 78 (GRP78) in bringing therapeutic benefits in AD models. In this study CM-695 a compound that selectively inhibits HDAC6 over class I HDAC isoforms and also inhibits phosphodiesterase 9 (PDE9) is used in chronic treatment of Tg2576 mice. Chronic treatment of TG2576 mice with CM-695 led to increase in chaperon protein HSP70 and GRP 78 which subsequently led to improvement in memory impairment and reduces brain Aβ (Cuadrado-Tejedor et al., 2019).

## Effects of HDACi in Various Neurodegenerative Disorders Models

### Spinal Muscular Atrophy

Spinal Muscular Atrophy (SMA) is a monogenic neuromuscular syndrome triggered due to mutations in Survival of motor neurone 1 (*SMN1*) gene (Maretina et al., 2018). The SMA patients maintain *in situ* copy of the paralog *SMN2* gene, which produces reduced amounts of the SMN protein. Therefore, the insufficient levels of SMN protein in motor neurons results into SMA. The increasing expression of SMN2 is a primary therapeutic approach for treatment of SMA (Sumner, 2006). Several studies have demonstrated potential HDAC inhibitors in treatment of SAM (Mohseni et al., 2013) Histone acetylation has been shown to upregulate SMA 2 expression (Kernochan et al., 2005). An analysis of differential regulation of SMA2 gene by silencing of individual HDAC isoforms by shRNA identified HDAC 2 and 6 as potential regulators of SMA 2 expression (Evans et al., 2011) Screening of a library of cyclic peptide inhibitors of HDAC on a neuronal cell line derived from a SMA patient's induced pluripotent stem cells identified HDAC inhibitors, which induced the expression of SMA2 (Lai et al., 2017). Sodium butyrate and its analog was shown to increase the expression of SMA2 in SMA cultured cells (Butchbach et al., 2016). Sodium butyrate also attenuated neurological symptoms in the mouse model of SMA (Minamiyama et al., 2004) In SMA models, SAHA has been shown to improve motor abilities, by improving the number of motor neurons in the mice. SAHA treatment was shown to improve weight loss in SMA mice (Riessland et al., 2010). SAHA was also shown to activate the SMA2 gene expression (Hahnen et al., 2006). In another study, daily injection of TSA, in SMA mice after the onset of disease lead to HDAC inhibition, triggered the *SMN2* gene expression and improved survival and motor pathology and attenuated weight loss (Avila et al., 2007). M344 a benzamide HDAC inhibitor showed highly strong (7 fold) upregulation of expression of SMA 2 gene in the fibroblast cells derived from a SMA patient (Riessland et al., 2006). A recent study reported that LBH589 treatment resulted in induction of H4 acetylation of SMN2 locus. A combinantion treatment of SMA cells with LBH589 and an antisense oligonucleotide that mimic Nusinersen (ASO_ISSN1) produced additive effects on SMN2 splicing and SMN protein expression (Pagliarini et al., 2019). Nusinersen, an anitisense oligonucleotide, has been recently approved by the US FDA for treatment of SMA (Hagenacker et al., 2020).

### Huntington Disease

Huntington disease (HD) is a rare genetic autosomal dominant neurodegenerative syndrome triggered by expanded CAG repeats in the huntingtin gene, which leads to pathological elongation of huntingtin with a polyglutamine tract. The neurodegenerative conditions caused due to HD lead to cognitive, motor, and psychiatric symptoms, atrophy of the basal ganglia and the cerebral cortex (Wright et al., 2017; Illarioshkin et al., 2018). Transcriptional dysregulation has been suggested a prominent mechanism for pathogenesis of HD (Sharma and Taliyan, 2015b). Hypoacetylation of histones associated with genes which are downregulation due to HD have been suggested for this dysregulation (Sadri-Vakili et al., 2007). The therapeutic ability of HDAC inhibitors have been demonstrated in different models of HD (Sadri-Vakili and Jang-Ho, 2006; Sadri-Vakili et al., 2007). Also, a genome wide decrease in H3 acetylation was demonstrated in a mouse model of HD (McFarland et al., 2012). Selective inhibition of HDAC6 increased acetylation of microtubules, which compensated for the transport deficit in HD (Vaughan et al., 2008). Reversal of H3 hypoacetylation with pimelic diphenylamine, an HDAC inhibitor, in transgenic HD mice improved the motor deficits (Thomas et al., 2008). Treatment with HDAC inhibitors in R6/2 a HD mouse model showed improvement in rotarod performance, which indicates improvement in motor impairment (Ellrichmann et al., 2017). SAHA showed its ability to penetrate the blood brain barrier and escalate histone acetylation in the brain (Hockly et al., 2003). (Ferrante et al., 2003) conveyed that sodium butyrate treatment attenuated neurodegenerative phenotype and enhanced survival in R6/2 HD models. SAHA and sodium butyrate treatment has been shown to retard neurodegeneration and improves lethality in a Drosophila model of HD (Steffan et al., 2001). An impaired microtubule transport is also attributed to the neuronal toxicity observed in HD. Vorinostat and TSA have been shown to increase vesicular transport of BDNF by inhibiting HDAC6 and thus increasing the acetylation of α-tubulin at Lys 40. Acetylation of α-tubulin at Lys 40 regulate the binding and motility of kinesin-1 (Dompierre et al., 2007). Treatment with RGFP966, a benzamide HDAC3 inhibitor, improved motor deficits in rotarod and in open field in N171-82Q transgenic HD mice. RGFP966 treatment caused induction in the expression of macrophage migration inhibitory factor, which is associated with activation of glial cells (Jia et al., 2016). LBH589, a non-selective hydroxamate HDAC inhibitor, induced histone hyperacetylation and prevented striatal neuronal shrinkage in R6/2 HD mice (Chopra et al., 2016). Interventional treatment in early presymptomatic phenotypes of HD with LBH589, an HDAC inhibitor, yielded significant improvements in behavioral changes (Siebzehnrübl et al., 2018). Characterization of interactome of endogenous HDAC4 in brains of HD mouse models revealed interaction of HDAC4 with Wiskott-Aldrich Syndrome Protein and SCAR Homolog (WASH) complex. This study confirmed the role of HDAC4 in progression of HD (Federspiel et al., 2019).

### Parkinson's Disease

Parkinson's disease is another neurodegenerative syndrome delineated by loss of dopaminergic neurons in the substantia nigra of the brain. HDACs and associated epigenetic functions have been suggested as potential therapeutic targets for PD (Harrison and Dexter, 2013; Sharma and Taliyan, 2015a; Hegarty et al., 2016b). A significant upregulation of HDAC 2 was reported in substantia nigra microglia of PD subjects (Tan et al., 2018).

In a study with a mouse model of PD treated with dopaminergic toxin MPTP, the administration of HDAC inhibitor phenyl butyrate significantly reduced the diminution of dopamine and loss of tyrosine hydroxylase-positive neurons in the substantia nigra (Gardian et al., 2004). Presynaptic α-synuclein mutations are linked with familial forms of PD (Gasser, 2001). The α-synuclein arbitrates neurotoxicity and targeting α-synuclein promotes toxicity, while cytoplasmic sequestration is protective in both cell culture and transgenic Drosophila (Kontopoulos et al., 2006). Toxicity caused by α-synuclein could be retrieved by the administration of HDAC inhibitors such as vorinostat and sodium butyrate in cell culture as well as transgenic flies. It was found that treatment with the HDACi lead to decreased neuronal death in response to α-synuclein (Kontopoulos et al., 2006).

In dopaminergic neuronal cells like MN9D, N27, and human SH-SY5Y cells, TSA treatment decreased survival rate and increased apoptosis, (Wang et al., 2009). This finding suggested that the effect of HDAC inhibitors might be complex. In some cases, the HDAC inhibitors could play a key role in pathogenesis of the neurodegenerative disease while in others these may act as feasible therapeutics for neurodegenerative disease. These contrasting effects of HDAC inhibitor may be attributed to varying epigenetic status, cell type and tissue specificity. HDAC inhibitors such as TSA and VPA, the pan-HDAC inhibitors of class I and class II are non-specific. Plausibly, this non-specificity might contribute to contradictory effects observed in different cell types (Wang et al., 2009). Neuron –restrictive silencer factor (NRSF) was suggested as a potential mediator for neuroprotective effects of TSA in MPTP induced model of PD (Suo et al., 2015). K560, a HDAC 1 and 2 isoform specific inhibitor, attenuated MPTP induced cell death in SH-SY5Y cells *in vitro* and prevented MPTP-induced loss of dopaminergic neurons in substantia nigra in mice *in vivo* (Choong et al., 2016). Similarly, M275 (HDAC 1 inhibitor) and tubastatin A (HDAC 6 inhibitor) improved sensorimotor reflexes and locomotor impairments (Pinho et al., 2016). The fibroblasts from PD patients with G2019S mutation in leucine rich repeat kinase 2 showed increased mitophagy and activation of class III HDACs (Yakhine-Diop et al., 2018). However, treatment with nicotinamide a class III HDAC inhibitor, though induced histone hyperacetylation and overexpression of neurotrophic factors but failed to provide neuroprotection. Nicotonamide treatment rather aggravated the PD pathology (Harrison et al., 2019). VPA was shown to protect SH-SY5Y dopaminergic neuronal cells from 6-OHDA-induced toxicity thorugh down regulation of apoptotic caspases namely, caspase-3, caspase-7, and caspase-9. VPA treatment also reduced the Bax/Bcl2 ratio in SH-SY5Y. This study suggested VPA treatment as a potential antidote for PD prevention (Hsu et al., 2020).

Most of the earlier reports on HDACi were focused on their anticancer, antiproliferative, and antiapoptotic properties. Hyperacetylation was associated with an elevation in expression of pro-apoptotic genes such as Bax and P21. Though, an escalation in acetylation also indorsed expression of anti-apoptotic or pro-survival genes like Bcl-2 and growth factors, which leads to protective effects in other cell types. This might be one mechanism by which HDAC inhibitors seem to be vindicatory in neurons and lethal in the cancerous cell (Saha and Pahan, 2005).

### Amyotrophic Lateral Sclerosis

Amyotrophic Lateral Sclerosis (ALS) is a neurodegenerative syndrome leading to progressive damage of motor neurons in the brain, and spinal cord causing weakness, muscle atrophy and death (Rowland and Shneider, 2001). The familial form of ALS is due to gain of functional mutation in the gene encoding Cu/Zn superoxide dismutase1 (SOD1) an antioxidant enzyme (Rosen et al., 1993). As transcriptional regulation may be involved in the pathophysiology of ALS, role of HDAC inhibitors have been studied in transgenic ALS mouse models. Target specific as well as global changes in histones acetylation are associated with ALS (Bennett et al., 2019). Global proteomic analysis suggested lysine-hyperacetylation of glial fibrillary acidic protein in post-mortem ALS spinal cord (Liu et al., 2013). Muscle HDAC 4 and its regulator microRNA-206 are upregulated in transgenic ALS mice carrying human mutations in SOD gene (Bruneteau et al., 2013). Analysis of differential expression of HDAC isoforms showed increased HDAC2 levels in ALS brain and spinal cord related to controls (Boeselt et al., 2010). Studies done by (Ryu et al., 2005) have shown that administration of 4-phenylbutryrate starting before or shortly after the beginning of symptoms resulted in increased survival, enhanced pathological phenotype and reduction in clinical impairment. VPA injection in G86R SOD1 mutant mice significantly attenuated the death of motor neurons, maintained the normal level of acetylation of histones and restored the loss of HAT CBP (Rouaux et al., 2007). Co- treatment of 4-phenylbutyrate and AEOL10150 a catalytic antioxidant to ALS mice had a collective effect on survival time and there was a reduction in the markers of oxidative damage in the lumbar spinal cord (Petri et al., 2006). Treatment of SOD-G93A transgenic ALS mice with TSA ameliorated muscle atrophy and neuromuscular junction denervation (Yoo and Ko, 2011). Role of HDAC 4 to mediate nerve-skeletal muscle interaction has suggested it as apotential target for ALS. Genetic deletion of HDAC4 in skeletal muscles caused earlier onset of ALS in a mouse model, suggesting a potential risk in use of HDACi for treatment of ALS (Pigna et al., 2019). A combinantion treatment with SAHA (a pan HDACi), RGFP109 (HDAC1/3 inhibitor), and arimoclomol attenuated the loss of nuclear FUS, a hallmark of ALS. HDAC inhibition also rescued the DNA repair response in iPSC-derived motor neurons carrying the ALS associated FUS^P525L^mutation. These observations suggest multiple mechanisms of neuroprotection by both HDAC inhibiting drugs and arimoclomol in ALS (Kuta et al., 2020).

### Alzheimer’s Disease

Alzheimer's Disease (AD) is well known for gradual memory loss and personality changes eventually preceding to dementia (Burns and Iliffe, 2009). The elevated level of extracellular β-amyloid (Aβ) and neurofibrillary tangles developing from *Tau* protein hyperphosphorylation are the neuropathological hallmark of AD (Luca et al., 2018). Impairments in HDAC functions and associated pathways have been implicated in pathogenesis of AD and HDAC inhibitors have shown promising applications for treatment of AD (Yang et al., 2017; Dubey et al., 2018). Different HDAC isoforms have been implicated in AD pathogenesis, loss of HDAC 5 was shown to impair memory function, while little effect on pathogenesis of AD in a mouse model for amyloid pathology (Agis-Balboa et al., 2013). Similarly, a decrease in HDAC6 levels re-established learning and memory and α-tubulin acetylation in a mouse model of AD. Further analysis suggested that HDAC6 loss rendered neurons resistant to amyloid-β-mediated deterioration of mitochondrial trafficking, an prominent factor for AD (Govindarajan et al., 2013). The levels of HDAC2 were found to be altered in the basal forebrain region containing cholinergic neurons of the Nucleus Basalis of Meynert in AD patients (Mahady et al., 2018). Overexpression of HDAC3 in the hippocampus increased Aβ levels, activated microglia, and decreased dendritic spine density in APPswe/PS1dE9 mice (Zhu et al., 2017). These studies have led to the advancement of pan and isoform selective HDAC inhibitors as potential therapeutic agents for treatment of AD (Yang et al., 2017).

Treatments with tubastatin A and ACY-1215 (specific HDAC6 inhibitors) improved behavioral loss, suppressed tau hyperphosphorylation and reduced amyloid-β (Aβ) load in AD mice. The treatments also resulted in elevated tubulin acetylation and reduced production and facilitated autophagic clearance of Aβ and hyperphosphorylated tau (Zhang et al., 2014). The daily administration of 4-phenylbutyrate in Tg2576 AD model reversed spatial memory deficit by regulating hyperphosphorylation of Tau in the hippocampus. However, the Aβ levels were not affected due to treatment with 4-phenylbutyrate (Ricobaraza et al., 2009). In another study, daily injection of low doses of valproic acid in Tg2576 AD mice at the early onset of 27 months, significantly reduced Aβ plaque number and improved memory deficit. The mechanism of Aβ plaque reduction by valproic acid was because of inhibition of GSK-3β-mediated γ-secretase cleavage of APP (Qing et al., 2008). Treatment of 3xTg-AD mice with a class III HDAC inhibitor, nicotinamide, prohibited memory impairment and reduced Tau pathology without affecting Aβ production. There was also a prolonged but little increase in internal p25 level, which was associated with improved learning and memory (Green et al., 2008). Treatment of Tg6799 AD mice with valproic acid in different disease stages (pre-, early- and late-symptomatic) caused a suppression in expression of nuclear factor kappaB (NF-κB) and IL-1ß in the plasma and increased NGF levels in the hippocampus. The treatments with VPA also decreased escape latencies of Tg6799 mice at early and late-symptomatic stages of AD (Noh and Seo, 2014). Chronic treatment with HDAC inhibitors sodium butyrate, valproate, or vorinostat showed the profound restoration of contextual memory in APPswe/PS1dE9 transgenic mouse model of AD. The newly established memories were firmly sustained over a 2-week period in HDACi-treated transgenic AD mice (Kilgore et al., 2010). Recently, inhibition of phosphodiesterase 5 (PDE5) and HDAC has been suggested as a potent novel therapeutic approach for AD (Rabal et al., 2016; Sánchez-Arias et al., 2017; Rabal et al., 2018). An acridine-based HDAC inhbitor, designed as a multi-target agent against HDAC and acetylcholine esterase showed selective inhbition of HDAC6 and strong activity against Aβ-aggregation (Tseng et al., 2020). A new tacrine-hydroxamate pan HDACi exhibited inhibitory activity on Aβ1-42 self-aggregation as well as disaggregation activity on pre-formed Aβ fibrils, the pathological markers of AD (Xu et al., 2020). Knockout of HDAC2 in mice leads to cognitive enhancement, and inhbitors of HDAC2 have shown potential as therapeutics for restotation of memory affected due to AD (Poplawski et al., 2020).

Acetylation of tau protein has been strongly asoociated with pathology of AD. Acetylation of tau has been shown to cause inhbition of its degradation leading to accummultaion of tau. A careful evaluation of tau acetylation has been suggested during treatment with HDACi (Jeong et al., 2019).

### Multiple Sclerosis

Multiple Sclerosis (MS) is an overwhelming autoimmune disorder of CNS. Recent findings have indicated towards the potential therapeutic role of HDACi in this disorder (SG and Dangond, 2006; Faraco et al., 2011). A significant increase in lysine acetylation was reported in myelin basic protein in an autoimmune encephalomyelitis (EAE) model of MS (Lillico et al., 2018). A study by Camelo et al. (2005) on EAE, a model of multiple sclerosis, demonstrated that neuropathology of mice with EAE was improved by TSA treatment. The animals treated with TSA showed less spinal cord inflammatory infiltrates, demyelination and axonal loss. Interestingly, TSA treatment also increased the number of motor neurons in the ventral horn, suggesting the potential application of TSA in neuroprotection. TSA treatment increased activity of neuroprotective proteins such as insulin growth factor-2 (IGF-2), estrogen receptor-α, glutamate transporter EAAT2 and glutathione peroxidase, and decreased the expression of pro-apoptotic Bax, Bid, caspase-2, and apoptosis-inducing factor (Camelo et al., 2005).

Axonal damage is also an important cause of neurodegeneration, where histone deacetylases play an important role. A study with a specific wallerian degeneration mouse (WID) shows delayed wallerian degeneration due to increase in activity of NAD^+^ dependent histone deacetylase SIRT 1. These mice had an elevated activity of the enzyme that produces NAD^+^ leading to increased SIRT 1 activity. The neuronal impairment due to progressive degeneration after axonal transfection in this specific mouse was inhibited (Araki, 2004). A recent study reported amelioration of clinical symptoms in the mouse model of MS due to loss of HDAC11 and suggested potential use of HADC-11 specific inhibitors for treatment of chronic progressive MS (Sun et al., 2018).

Among the six animal models of neurodegenerative disorder described above treatments with different class of HDAC inhibitors shows beneficial effect of HDAC inhibitors in improving neurodegenerative conditions by improving survival of neurons and preventing neuronal death through different mechanism (**Table 2**).

**Table 2:** HDAC Inhibitors used in neurodegenerative disease models and their effect.

		HDAC inhibitors used	Effect of HDAC inhibitors treatment	References
**Neurological Disease Models**	**ALS**	Valproic acid, Trichostatin A 4-Phenyl Butyrate 4-Phenylbutyrate + Antioxidant Valproic acid + Lithiun 4-Phenybutyrate + Riluzole	Reestablished CBP loss and histone hypoacetylation Suppressed motor neuronal death Improved motor function and survival of neurons	Sugai et al., 2004; Ryu et al., 2005; Petri et al., 2006; Rouaux et al., 2007; Del Signore et al., 2009; Yoo and Ko, 2011
	**AD**	Sodium butyrate, Vorinostat 4-phenylbutyrate, Valproic acidNicotinamide, Tubastatin A ACY-125	Reestablished hypo acetylation Decreased Aβ plaque number and improved memory deficit Enhanced tubulin acetylation, reduced production and facilitated autophagic clearance of Aβ and hyperphosphorylated tau Reversed spatial memory deficits	Fischer et al., 2007; Green et al., 2008; Kim et al., 2008; Qing et al., 2008; Guan et al., 2009; Ricobaraza et al., 2009; Noh and Seo, 2014; Zhang et al., 2014
	**SMA**	Vorinostat, 4-Phenylbutyrate Nicotinamide	Increased SMN2 expression Increase histone acetylation Improved survival and motor pathology Increased induced Bcl-2, Bcl-XL and BDNF	Chang et al., 2001; Brichta et al., 2003; Sumner et al., 2003; Andreassi et al., 2004; Hahnen et al., 2006; Avila et al., 2007; Hauke et al., 2009; Riessland et al., 2010
	**HD**	Vorinostat, Trichostatin A Sodium Butyrate 4-Phenylbutyrate HDACi 4b, Tubacin RGFP966 + Pimelic diphenylamine LBH589	Normalized striatal atrophy and degeneration Restored histone hypoacetylation and transcriptional dysfunction Increased vesicular transport of BDNF and improved motor performance and survival	Steffan et al., 2001; Ferrante et al., 2003; Hockly et al., 2003; Bates et al., 2006; Dompierre et al., 2007; Sadri-Vakili et al., 2007; Pallos et al., 2008; Thomas et al., 2008
	**PD**	Valproic acid, Tubastatin A 4-Phenyl Butyrate, M-275 Sodium Butyrate, Trichostatin A	Reduction in dopaminergic death Increased acetylation of α-tubulin Increased GDNF and BDNF expression Improved sensorimotor reflexes and locomotor impairments	Gardian et al., 2004; Chen et al., 2006; Kontopoulos et al., 2006; Wang et al., 2009; Lu et al., 2013; Wu et al., 2013; Choong et al., 2016; Pinho et al., 2016
	**MS**	Trichostatin A, ITF2357 SAHA	Reduction in spinal cord inflammatory infiltrates, demyelination and axonal loss increase in number of motor neurons in the ventral horn Improvement in neuronal pathology	Camelo et al., 2005; Lillico et al., 2018; Sun et al., 2018

HDAC, Histone deacetylases; ALS, Amyotropic Lateral Sclerosis; AD, Alzheimer's disease; SMA, Spinal Muscular Atrophy; HD, Huntington's disease; PD, Parkinson's disease; MS, Multiple Sclerosis; SAHA, suberoylanilide hydroxamic acid.

## Neuroprotective Effects of HDACi

Potential of therapeutic agents to generate neuroprotective actions has been considered as an important factor for their development for treatment of neurodegenerative disorders. Beside potential for treatment of neurogenerative diseases, the HDAC inhibitors have also been reported to produce prominent neuroprotective properties.

VPA has been shown to reduce the retinal neuronal death by optic nerve crush (ONC). VPA up- regulated levels of BDNF, Bcl-2, and TrkB in the retina post-injury (Zhang et al., 2012). VPA and Sodium butyrate treatment caused hyperacetylation of histone H3K14, reduced histone H3K9 hypermethylation in the BDNF promoter and increased transcriptional activity. These results suggest that VPA seemed to protect retinal ganglion cells (RGC) from ONC by hindering neuronal apoptosis, presumably, *via* the initiation of HDAC inhibition and BDNF-TrkB signaling (Zhang et al., 2012). BDNF gene expression has also been associated with mood-stabilizing effects in bipolar disorder (BD). BDNF plays an important role in promoting neuronal survival and synaptic plasticity and well-studied as a biomarker of illness activity in BD (Kuipers and Bramham, 2006). (Stertz et al., 2014) examined repeated exposure of D-amphetamine (AMPH) in a bipolar animal model and also investigated the effect of sodium butyrate. They found that repeated AMPH administration increased the HDAC function in the prefrontal cortex (PFC) without changing BDNF protein or mRNA levels, while the administration of sodium butyrate partially reversed the HDAC activity. HDAC inhibitors have shown potential for therapeutic application for treatment of neurodegenerative and psychiatric disease having neuroprotective and memory enhancing properties and increased expression of BDNF. (Koppel and Timmusk, 2013) compared the effect of class I, I/IIb, and II selective HDAC inhibitors on BDNF mRNA expression in rat primary neurons. The results indicated that inhibition of class II HDACs led to rapid elevation of BDNF mRNA levels, while class I HDAC inhibition created a distinctly delayed BDNF induction. Their finding suggested that class II HDAC inhibitors play an important part in regulating transcription of BDNF and may have potential application for treatment of neurological disorders. (Hasan et al., 2013) investigated the role of HDAC inhibitors in neuroprotection after ischemia. Their study demonstrated that VPA and TSA treatment after oxygen and glucose deprivation led to re-oxygenation in rat cortical neurons, promoted neuronal regeneration and neuronal protection through upregulation of BDNF expression. There was also a significant increase in three synaptic markers, namely PSD95, GAP 43, and synaptophysin in VPA and TSA treated rat cortical neurons. (Chen et al., 2006) have shown that VPA promoted dopaminergic neuron in midbrain neuron/glial culture by stimulating the release of neurotrophins like BDNF and GDNF. VPA pretreatment also protected midbrain dopaminergic (DA) neurons from LPS or 1-methyl-4-phenylpyridinium (MPP^+^)- induced neurotoxicity. LPS treatment impaired the uptake process of DA by 45% and this decrease was strongly hindered by VPA pretreatment in a dose-dependent manner. Similarly, treatment with MPP^+^ also reduced DA uptake by 70% and VPA significantly reduced the degeneration of DA neurons by MPP^+^ toxicity. A recent study by (Lu et al., 2013) with LB-205 (a pan HDAC inhibitor) in acute traumatic brain injury rat model showed that LB-205 treatment increased the survival of central nervous system cells after the injury by virtue of preservation of NGF expression and activation of tyrosine kinase A receptor (TrKA) pathway. The neuroprotective mechanism of HDACi was linked with upregulation of expression of NGF, activation of TrkA phosphorylation, phospho-protein kinase B (p-AKT), NFκB, and B-cell lymphoma 2 (Bcl-2) cell survival factors, while the downregulation of phospho-JNK, p75 neurotrophin receptor (NTR), and Bcl-2-associated X protein apoptosis factors (Wu et al., 2013) studied the effect of VPA, TSA, 4-phenyl butyric acid (4-PBA), and nicotinamide in astrocyte and dopamine neuron glial culture. TSA and VPA increased GDNF and BDNF transcripts in astrocytes. The neurotrophin transcript levels were increased due to increase in GDNF promoter activity leading to elevation in level of H3 acetylation. VPA, TSA, 4-PBA, and nicotinamide also protected dopamine neuron/glial culture from MPTP and LPS toxicity. Both 4-PBA and nicotinamide decreased the release of TNF-α and cell survivability in microglial culture stimulated with LPS (Wu et al., 2013).

Treatment of SH-SY 5Y cells with VPA, motivated neurite outgrowth and cell survival by triggering ERK pathway. VPA treatment enhanced norepinephrine uptake and also induced expression of GAP-43 and Bcl-2, the genes controlled by ERK pathway (Yuan, 2001). VPA has been shown to prolong the life span of cultured cortical neurons and promote neuronal growth (Hao, 2004). *In vivo* effects have also shown HDAC inhibitors to be neuroprotective in chronic neurodegenerative diseases (Siebzehnrubl et al., 2007). Increased histones acetylation and the transcription factors in neurons have been linked to shield from apoptosis in animal models of neurodegeneration and promote neuronal differentiation (Siebzehnrubl et al., 2007). Both, TSA and VPA have been linked to induce differentiation of hippocampal neural progenitor cells (Hsieh et al., 2004). HDAC inhibitors like MS-275, M334, and SAHA increased neuronal differentiation and inhibited oligodendrocytes production in adult subventricular zone precursor cells (Siebzehnrubl et al., 2007). (Zhou et al., 2011), showed that SAHA and sodium butyrate blocked cell cycle progression from G1 to S in the neurosphere formation of adult subventricular cells. Pro-neural transcription factors such as Neurog1 and Neurod1 were up regulated. SAHA has also been found to be neuroprotective in dopaminergic neurons from midbrain primary culture by induction of neurotrophic factors from astroglia through induction of histone acetylation (Chen et al., 2011). (Jung et al., 2008) have reported VPA to stimulate differentiation and reduce proliferation in rat cerebral cortex neural progenitor cells *via* the activation of ERK-P21 Cip/WAFI pathway. HDAC inhibitors have also shown roles in directing neuronal, cardiomyocytic and hepatic lineage differentiations (Kretsovali et al., 2012).

SAHA treatment increased survival of dopaminergic neurons in mesencephalic neuronal culture, which was evident by an increase in percentage of dopamine uptake (Chen et al., 2011). The neuroprotective quality of SAHA was attributed to astroglia. SAHA was also found to induce the release of neuronal survival factors from astroglia by increasing the release of neurotrophic factors GDNF and BDNF. SAHA induced hyperacetylation of histones and also the induced expression of GDNF and BDNF in mesencephalic neuronal culture (Chen et al., 2011). A study on the effect of various HDAC inhibitors on survival of rat retinal ganglion cells (RGC) led to the finding that sodium butyrate and VPA delayed the spontaneous cell death in RGC. However, TSA did not have the same effect in these cells (Biermann et al., 2011).

## HDAC Inhibitors in Neuronal Differentiation

HDAC inhibitors induce histone acetylation in diseased neurons and may reinstate transcriptional stability and hence delay or prevent cell degeneration (Sterner and Berger, 2000). The neurotrophic activities of HDAC inhibitors may be epigenetically controlled by acetylation of histones along with non-histone proteins such as transcription factors (Sterner and Berger, 2000). The regulation of transcription by epigenetic modifications has been established to be significant for neurite outgrowth and neuroprotection in the peripheral nervous system during neuronal development. The increased acetylation of both transcription factors and histones in neurons has been revealed to stimulate neuronal differentiation and outgrowth (Gaub et al., 2010). TSA has been shown to produce differentiation of retinal ganglion Cells (RGC-5 cells). The differentiation of RGC-5 cells induced by TSA was transcription dependent, as the neurite outgrowth was blocked in presence of a RNA polymerase inhibitor alpha amanitin (Schwechter et al., 2007b). The differentiation of RGC-5 cells with HDAC inhibitors might be due to elimination of neuron gene-specific transcription repressor—neuron restrictive silencing factor (NRSF) from NRSE. It is critical process, however, not essentially adequate component of *in vivo* neuronal differentiation (Schwechter et al., 2007b). (Tomioka et al., 2014) have shown that TSA induced neurite outgrowth in PC12 cells by an expression of nur77, which was upregulated due to acetylation of Lys 14 on histone H3. (Wang et al., 2014) have shown that TSA improved the expression of neurotrophic factors in the Schwan cells (SC) and repressed the expression of genes associated with myelin production. TSA indicated to play a key role in the growth and biological functions such as proliferation, survival, migration, and myelination of SCs (Wang et al., 2014). In cultured cortical granule neuron (CGN), hyperacetylation stimulates neuronal outgrowth and growth cone remodeling through inhibition of HDAC I and HDAC II by a transcription-dependent mechanism. It was found that TSA induced a significant increase in expression of HATs such as p300, CBP and P/CAF in cultured CGN. Acetylation dependent p53 signaling functions are also important events, which allow physiological pro-outgrowth signal in primary neurons (Gaub et al., 2010). Transcriptional control, chromatin remodeling, and epigenetic modifications regulate the self-renewal and differentiation of neuronal stem cells (NSC) (Temple, 2001). Throughout the growth of the central nervous system in vertebrates, the fate of NSC is firmly controlled under temporal and regional manners and is also regulated by epigenetic controls (Temple, 2001). Reports have indicated that the HDACs play key role in regulation of proliferation and differentiation of neural stem cells (Sun et al., 2011). HDAC 1, 3, 5, and 7 are expressed in neural stem cells. However, the expression of these HDACs is significantly reduced after differentiation (Sun et al., 2007). HDAC2 is expressed in proliferating neural stem cells and is up regulated as neurons differentiate (MacDonald and Roskams, 2008). A study on the effect of TSA on multipotent neural stem cells, that have the ability for self- renewal and differentiation into neurons astrocytes and oligodendrocytes, demonstrated that TSA dose-dependently, promoted neurite outgrowth in multipotent neural stem cells by significantly increasing branching points and dendritic area. However, TSA inhibited the differentiation of NSC into astrocytes. TSA also mediated cholinergic differentiation of NSC as evident by a 10% increase in cholinergic neurons after TSA treatment (Balasubramaniyan et al., 2006). HDAC inhibitors have also been shown to increase neuronal differentiation of embryonic mouse neural stem cells (Siebzehnrubl et al., 2007). VPA treatment, at the progenitor stage of hippocampal treatment, led to robust inhibition of cell proliferation and stimulation of neuronal differentiation due to an increase in expression of proneural transcription factor. The treatment with HDAC inhibitors increased expression of Ngn, Math1, and P15 and shifted the cells towards neuronal fate. Also, the increased expression of proneural transcription factor was linked to acetylation of histone H4 (Yu et al., 2009). Similar to neural stem cells, the pluripotent stem cells have also shown the potential for the treatment of neurodegenerative disease owing to their competency to self-renew and differentiate into all cell types (Thomson, 1998). Pluripotent stem cells (iPSCs) are alike embryonic stem cells and are derived from epigenetic reprogramming of somatic cells by the exogenous expression of pluripotency-related transcription factors (Hochedlinger and Plath, 2009). HDAC inhibitors improve the reprogramming efficacy of the somatic cells into pluripotent cells (iPSC). HDAC inhibitors like, SAHA, TSA and VPA have shown an important role in reprogramming of mouse embryonic fibroblast to iPSCs (Huangfu et al., 2008). HDAC inhibitors have also been shown to play an important role in survival, proliferation, and differentiation of rat neural precursor cells (NPC). TSA treatment of NPC derived from Sprague Dawley rats E14.5 brain has shown decreased proliferation and increased differentiation of neurons along with decreased differentiation of astrocytes. Increased neuronal differentiation and proliferation of NPC was attributed to reduction in transcription of class II HDAC, while the transcription of class I HDAC was not changed (Liu et al., 2012). Hisahara et al. (2008) have reported important role of SIRT1, a NAD+ dependent class III HDAC in the differentiation of NPC. The SIRT1 was found predominantly in cytoplasm. However, when cultured NPCs were transferred to differentiation conditions, SIRT1 was expressed in the nucleus. This implicated modulation of neuronal differentiation by SIRT1 nuclear translocation. SIRT1 plays an important role in neuronal differentiation as evident by decrease in neuronal differentiation by inhibition of SIRT 1 by sirtinol, a SIRT1 inhibitor as well as use of siRNA against SIRT1. Overexpression of SIRT1 promoted neuronal differentiation (Hisahara et al., 2008). VPA induced neuronal differentiation in adult hippocampal neural progenitor cells (Hsieh et al., 2004). VPA treatment also increased the level of acetylated histones. The level of acetylated histones was elevated in the neuronal cells as compared to astrocytes and oligodendrocytes. The acetylation of histones promotes the differentiation of adult hippocampal progenitor cells; however, deacetylation of histones or deacetylase activity enables differentiation of oligodendrocytes and astrocytes. The VPA induced neuronal differentiation of neural progenitor cells was linked to upregulation of a neuron specific gene NeuroD, a bHLH transcription factor (Hsieh et al., 2004). In another study, VPA produced differentiation of cortical neurons by activation of ERK Pathway. Valproate induced phosphorylation of P44/42 ERK were MEK and REF dependent, which was similar to action of neurotrophins (Hao, 2004). VPA treatment in rat cerebral cortex induced differentiation and reduced the proliferation of neural progenitor cells *via* activation of ERK-P21 Cip/WAFI pathway mediated by β-catenin pathway (Jung et al., 2008). (Marin-Husstege et al., 2002) developed two pronged approaches to investigate the role of chromatin remodeling in oligodendrocyte differentiation. The first approach assumes chromatin compaction on negative regulatory sites of a differentiation gene. While, the second or alternative approach proposes chromatic compaction in the promoter of a gene encoding inhibitors of differentiation and in turn blocking the access to transcription start site. Histone deacetylation has also been shown to be important for oligodendrocyte differentiation. Deacetylation followed by methylation of histones promoted the neural stem cells differentiation into neurons and glial cells. The HDAC activity was vital for differentiation of oligodendrocyte from rat cortical progenitor cells (Marin-Husstege et al., 2005). Investigation on the effect of VPA on neuronal differentiation with human bone marrow-mesenchymal stromal cells (hBM-MSCs) showed that VPA treated MSCs differentiated on neuronal media and showed an increase in number/length of neurites as compared to differentiated MSCs. The VPA treated MSCs exhibited cell morphology similar to regular neurons. There was a significant increase in expression of neuronal marker such as Nestin, Musashi, CD133, and GFAP (Jeong et al., 2013). Sodium butyrate enhanced neuronal differentiation of medulloblastoma (MB) cells, the malignant brain tumors of childhood. Sodium butyrate triggered the Gria2 expression, a neuronal differentiation marker in human medulloblastoma cell lines, D283 and DAOY. HDAC inhibitors have shown to reduce MB stem cells survival and proliferation (Nör et al., 2013). VPA precisely escalated neuronal differentiation in human *FGF1* gene 1B promoter (-540 to +31) driven green fluorescence F1BGFP (+) embryonic stem cells and Neural stem/progenitor cells (NSPCs) rather than GFP(-) cells (Kao et al., 2013). These NSPCs were crucial for VPA-induced neuronal differentiation than GFP (-) cells. VPA activated human *FGF1* gene promoter by dissuasion of HDAC and GSK-3 functions. (Her et al., 2009) have shown that HDAC inhibitors, namely TSA, sodium butyrate, and VPA, promoted differentiation of rat C6 glioma cells by producing neuroactive 5α-reduce neurosteroid, which stimulated expression of serotonin stimulated brain derived neurotrophic factor transcription. These HDAC inhibitors also stimulated production of glial fibrillary acid (GFAP).

Niemann–Pick type C disease (NPC) is a kind of neurodegenerative condition associated with lipid storage disorder (Liscum and Klansek, 1998). In NPC1-/-, mice self-renewal, and differentiation of neural stem cells (NSC) are impaired. Treatment of NPC1-/- mice with VPA enhanced neuronal differentiation and restored impaired astrocytes in NSCs from NPC1^−/−^ mice. There was an upregulation of vital neurotrophic genes (*BDNF*, *MnSoD*, *TrkB,* and *NeuroD*), by suppression of the REST/NRSF and HDAC complex by VPA treatment. Up-regulated neurotrophic genes enhanced neural differentiation in neural stem cells from NPC1^−/−^ mice (Kim et al., 2007).

HDAC1 has also been shown to have an important player in regulating the switch between proliferation and differentiation in the retina of zebra fish. The ratio of differentiating cells to that of proliferating cells was increased proportionate to HDAC activity due to treatment of zebrafish with TSA, which suggests that HDAC might control the retinal neurogenesis in zebrafish (Yamaguchi, 2005).

## HDAC Inhibitors as Potential Neurotrophins

Neuroregeneration is a new notion that includes neuroplasticity, neurogenesis, and neurorestoration (Enciu et al., 2011). Earlier, neuronal regeneration in the mammalian central nervous system (CNS) was considered to be untreatable, but recent reports have suggested that damaged neurons can be restored with stimulatory substances for example nerve growth factor (NGF) and brain derived neurotrophic factor (BDNF) (Diaz Brinton et al., 1998; Filbin, 2000; Fournier and Strittmatter, 2001; Pesavento et al., 2002). Neurite outgrowth, an important cellular process for neurogenesis, is a complex three stage process 1) sprouting; neurite formation initiation, 2) axon elongation, and 3) dendrites branching after the formation of synapses (Kiryushko et al., 2004). Neuritogenesis is a key cellular differentiation process for formation of new neurons. The cellular and molecular mechanisms of neuritogenesis is important, as it is essential for proper brain wiring and nerve regeneration, and along with various neurodegenerative diseases (Jones et al., 2001). Recent reports have emphasized the importance of neurite outgrowth in neuroregeneration and damaged neuronal repair (Tomioka et al., 2014). The physiological substances, which trigger the process of neuritogenesis, are commonly referred as neurotrophic factors. Neurotrophic factors are proteinaceous growth factors that stimulate neurite outgrowth and repair damaged neurons. It may also play a key role in regression and reestablishment of the damaged neurons to normal neuronal functions (Li et al., 2002). These factors are produced and released by the target neuronal tissues and initiate the growth and differentiation of developing neurons. The NGF, BDNF, neurotrophin-3 (NT-3), and neurotrophic factor-4/5 (NT-4/5) are commonly referred as neurotrophins. Use of natural neurotrophins appears to be an obvious choice for neuronal regeneration. However, regardless of their definite positive effects, therapeutic applications of natural neurotrophins like NGF and BDNF, for treatment of neurodegenerative diseases, are limited due to their inability to cross blood brain barrier and also susceptibility to degradation by peripheral peptidases (Tomioka et al., 2014). Recently, there has been significant surge in identification of small molecular weight neurotrophic compounds, which can promote growth, differentiation, survival of developing and damaged neurons (Longo and Massa, 2013; Obianyo and Ye, 2013). Several studies have been performed using neurotrophins as a probable therapy for neuronal degeneration and trauma causing severing of nerve connections. The neurotrophins may facilitate the survival of physically damaged neurons like injury by motorcycle accidents or sports-related injuries or in neurodegenerative diseases like Huntington's, Alzhiemer's, and Parkinson's disease. The therapeutic use these neurotrophins offered hope to make a more widely accepted clinical possibility for neuroregeneration. Several recent finding indicates that neuroprotective and neurotrophic role of small molecular weight inhibitors of HDACs and their possible use in treatment of neurological disorders (Camelo et al., 2005; Xu et al., 2011). HDAC inhibitors by chromatin modification and epigenetic regulation provides unique neuronal acclimatization like transcription-dependent neuroplasticity and learning or axon and dendrite regeneration (Kandel, 2001; Wu et al., 2007). Vorinostat exhibited independent neurotrophic action on NS-1 cells, clone of PC12 cells. Interrogation of intracellular neurotrophin signaling pathways with selective inhibitors of MEK1/2, phosphoinositide 3-kinase (PI3K) and tyrosine kinase A (TrkA) suggested the role of ERK pathway in vorinostat-induced neurotrophic action also involved the activation of upstream extracellular kinase TrkA. Vorinostat (SAHA) stimulated hyperacetylation of α-tubulin and histones H3/H4 in NS-1 cells (Shukla et al., 2016). CTPB [N-(4-chloro-3-trifluoromethyl-phenyl)-2-ethoxy-6-pentadecyl-benzamide], an activator of the histone acetyltransferase p300/CBP also showed neurotrophic action on in the SH-SY5Y neuronal cells. This report confirms the role of proteins hyperacetylation in induction of neurotrophic signaling pathways (Hegarty et al., 2016a). Treatment with VPA caused hyperacetylation of histones and elevated levels of neurotrophic/neuroprotective factors, which provided neuroprotective and neurorestorative phenotype in an *in-vitro* model of PD (Harrison et al., 2015). VPA treatment also induce neurotrophic factors in neuronal stem cells (Almutawaa et al., 2014). The neurotrophic action of TSA in a rat cultured cerebellar granule neurons and in cerebral cortical neurons involved acetylation of p53 and also protected neurons against glutathione depletion-induced oxidative stress (Maruoka et al., 2011). Treatment with sodium butyrate stimulated neurogenesis in the ischemic adult rat brain and also elevated protein levels of phospho-CREB, BDNF, and glial fibrillary acidic protein. The neurotrophic actions of sodium butyrate were blocked by intraventricular injection of K252a, a tyrosine kinase B receptor antagonist. (Kim et al., 2009). *In vitro* treatment of the retinal ganglion cell line (RGC-5) with TSA showed significant RNA transcription-dependent neurotrophic action (Schwechter et al., 2007a). The prominent neurotrophic potential of HDAC inhibitors strengthens their therapeutic application for treatment of neurodegenerative disorders.

## Conclusion

This review presents the current status of knowledge about the role of various HDAC inhibitors in neuronal differentiation and neuroprotection in different cell lines, *in vivo* preclinical animal models and limited information on their clinical evaluations. The potential therapeutic application of several HDAC inhibitors for neurodegenerative diseases has also been discussed. HDAC inhibitors have significant therapeutic potential for many of the neurodegenerative diseases such as PD, AD, SMN, ALS, TBI, MS, and HD. HDAC inhibitors engender neuronal differentiation in neural stem cells (NSC), neural progenitor cells (NPCs), rat retinal ganglion cells (RGCs), dopaminergic neurons, SHSY5Y neuroblastoma cells, cortical granule neurons (CGN) and experimental cell lines such as the PC12 cells. HDAC inhibitors also possess neuroprotective and neurotrophic potential. However, potential limitations regarding safety of these HDAC inhibitors cannot be ignored. Reports indicate that HDAC inhibitors in some instances could play a role in pathogenesis of the neurodegenerative disease, while in others could act as potential therapeutics for the neurodegenerative disease. These opposing effects of HDAC inhibitors could be attributed to the varying epigenetic status of the targets, cell type and tissue specificity. On occasion, the HDAC inhibitors have been shown to be highly neurotoxic rather than being neuroprotective. The major problem associated with HDAC inhibitors is their lack of specificity and in particular their lack of isoform selectivity. Another issue is incomplete knowledge about the off-target actions of HDAC inhibitors. There is a great surge for learning about the mechanism of action and the various ways that HDAC inhibitors affect gene expression. Future research is expected to elucidate the mechanism of action HDAC inhibitors in order to design novel reagents with increased effectiveness and specificity. Research on specific/selective HDAC inhibitors would be beneficial from the therapeutic point of view and would likely overcome the problems currently associated with HDAC inhibitors. Hopefully, the rapid interest in the study of HDAC inhibitors will shed some light on the mechanism of action of these inhibitors and give us directions to combat neurodegenerative diseases.

## Author Contributions

SS and BT conceived the article. SS compiled the review and prepared the draft of the manuscript. BT reviewed and edited the manuscript. All authors read and approved the manuscript.

## Conflict of Interest

The authors declare that the research was conducted in the absence of any commercial or financial relationships that could be construed as a potential conflict of interest.
